# Supplementary material for: Pathways of health care for people living with multimorbidity in two Southern African countries
Source: PLoS One. 2026 Jun 12;21(6):e0351251. doi: 10.1371/journal.pone.0351251 (PMC13262806; doi:10.1371/journal.pone.0351251)
Supplement: S1 Table — (DOCX) [file pone.0351251.s002.docx]

**S1 Table : Table summarising the included policy documents**

| **Document Title** | **Years** | **Conditions** | **Intended Purpose** | **Comment** |
| --- | --- | --- | --- | --- |
| **Malawi [1]** | | | | |
| **Health Promotion Policy** | 2013 | HIV, hypertension, diabetes, and mental health. | To reduce preventable deaths and disability through effective health promotion interventions. | The policy focuses on service provision within the primary health sector. It mentions the need for health promotion to prevent morbidity and mortality |
| **National HIV Prevention Strategy** | 2015-2020 | HIV, NCD (NCDs mentioned briefly). | Reduce new HIV infections by 70% by 2020/reduce stigma and discrimination. | The policy aimed to guide the formulation of integrated NCD and HIV clinical guidelines. |
| **National Alcohol Policy** | 2017 | HIV, cancer, diabetes, heart disease, liver cirrhosis. | To promote health sectors' response to harmful alcohol use and alcohol related diseases |  |
| **National Action Plan for the Prevention and Management of Noncommunicable Diseases in Malawi** | 2017-2022 | cardio-vascular diseases, diabetes, cancer, chronic kidney diseases, chronic lung diseases and chronic neurological diseases (e.g., epilepsy, mental illnesses). | Improve the level of sustained, accessible, comprehensive, and integrated chronic NCD services. | Called for NCD services that incorporate prevention, early detection and screening, diagnosis, referral, and treatment. |
| **National Health Policy** | 2018 | HIV/AIDS, cardiovascular diseases/NCDs. | Reduce premature mortality from NCDs by 1/3 by 2030; to combat the HIV epidemic. | The policy gives general direction for achieving UHC for Malawians, with a mention of those living with HIV and NCD as separate disease groups. |
| **Malawi National Cancer Control Strategy** | 2019-2029 | cancers, HIV. | Reduce the incidence, morbidity, and mortality and improve the quality of life for people living with cancer | The strategy calls for integrated HIV and cancer services, but acknowledges the lack of prevalence data on Cancers in Malawi |
| **National Strategic Plan for HIV/AIDS** | 2020-2025 | HIV, NCD. | To increase coverage and provision of high-quality integrated HIV and other related diseases (NCD, Viral Hepatitis, and cancer services); Screen and effectively treat NCDs in People Living with HIV, especially hypertension, diabetes and dyslipidaemia. | This policy promotes integrating HIV with TB, NCDs, Viral Hepatitis, mental health and Nutrition. |
| **Malawi Mental Health Policy** | 2020 | NCDs, HIV, “chronic conditions”, epilepsy, mood affective disorders. | Reduce the burden of mental health problems and improve access to integrated quality mental health services. | The policy mentions the association between mental ill health and HIV/AIDS, but does not mention integrating mental health and other NCDs. |
| **Malawi PEN-PLUS Operation Plan** | 2021 | Hypertension, diabetes, chronic kidney disease |  | This outlines care for each healthcare level and lays out referral systems for NCDs |
| **Health Sector Strategic Plan 3** | 2023-2030 | HIV/AIDS, hypertension (CVD), diabetes, chronic respiratory disease, cancer, neurological and mental health disorders, injuries and emergencies. | To improve the availability, accessibility and quality of health infrastructure and medical equipment at all levels of health care; to improve the availability of competent and motivated human resources for health for quality health service delivery that is effective, efficient and equitable. | The document promotes integration at all levels of the health system, from budgeting to service delivery. |
| **Zimbabwe** | | | | |
| **Zimbabwe National Health Strategy 2021-2025** | 2021 | Chronic infectious diseases (HIV/AIDS and TB), NCDs (including hypertension, diabetes, chronic respiratory disease, cancer, neurological and mental health disorders), and injuries and emergencies | Sets out the strategic direction for the health sector over the next five years in order to attain health equity for all. Has increased emphasis on non-communicable diseases as a major emerging cause of morbidity and mortality amongst across socio-economic strata. Aims to reduce mortality from cervical cancer from >20% to <5% by 2025 (only NCD indicator) | A salient document that provides lucid recognition of the persistence of siloes in the health sector, the uneven prioritisation of HIV and TB relative to NCDs, and the need for considerable action to improve the latter. NCD data to guide action still heavily reliant on 2005 STEPS survey, highlighting the need for improved data as a key step towards improving awareness. |
| **Zimbabwe National Development Strategy 2021-2025** | 2021 | Communicable and NCDs, nutrition and healthy lifestyles, and access to safe environments. | Aims to provide steps towards Zimbabwe becoming an upper middle  income society by 2030. Health framed as a key pillar of economic development, calling for greater domestic health funding. Aiming to increase per capita spending from $30.29 per capita in 2020 to USD$86 by 2025. | Provides a good foundation for seeing how health figures within the broader development agenda of Zimbabwe. Key programmes mentioned include HIV and TB and the prevention and control of NCDs, reflecting growing recognition of the huge burden these conditions now pose. |
| **Zimbabwe National HIV and AIDS Strategic Plan 2021-2025** | 2021 | HIV/AIDS and certain HIV-associated comorbidities including hypertension, diabetes, and mental health | Provides the strategy for the next five years on the prevention, care and treatment of HIV aiming to achieve 95-95-95 while ensuring quality of care through a differentiated, person-centred care approach operating from community to quaternary level. HIV-NCD integration a new pillar within the strategy | NCD integration into HIV services now recognised as a core pillar of the HIV response amidst the ‘greying’ of HIV. Still only a limited number of NCDs included, and in general the priority for multimorbidity is mostly in the way that it impacts the ability to meet HIV targets rather than specifically prioritising NCDs and multimorbidity. |
| **Operation and Service Delivery Manual for the Prevention, Care and Treatment of HIV in Zimbabwe** | 2022 | HIV/AIDS and certain HIV-associated comorbidities including hypertension, diabetes, and mental health. | Provides comprehensive operational guidance for health workers from doctors to community health workers on the prevention, care and Treatment as a complement to the National HIV prevention, testing and treatment guidelines | Provides a window into how the health workforce operationalises HIV guidance and broader programmatic efforts. Provides steps on the ‘hows’ of HIV care, including the recent addition of NCDs into service integration. |
| **Zimbabwe National Survey**  **Zimbabwe Non-Communicable Disease Risk**  **Factors - (ZiNCoDs)**  **Preliminary Report** | 2005 | Risk factors for common NCDs, including hypertension and diabetes. | The last national survey on NCD risk factors was conducted over 20 years ago to provide representative data on NCD risk factors and the prevalence of key NCDs. Remains a key citation in most policy documents around NCDs, including the National Health Strategy. | Very outdated but still heavily cited as the last representative survey of NCDs in Zimbabwe. All other knowledge comes from country profiles based on modelling and other limited data sources |
| **Zimbabwe national cancer control strategy 2025-2030** | 2025 | All cancers, but priority given to breast, prostate, colorectal, and childhood cancers | Provides the strategy for the next five years for the prevention and control of cancers in Zimbabwe. Core pillars of the strategy are scaling up and improving quality of services, strengthening the workforce, ensuring adequate equipment and infrastructure, improving M&E, and strengthening leadership and governance. | Has been consistently updated since the mid-2000s, though cancer remains extremely difficult to manage due to resource scarcity. Many of the gains have come in the context of cervical cancer, which forms part of the relatively well-funded HIV programme. |
| **Zimbabwe National Healthy Ageing Strategic Plan**  **2017-2020** | 2017 | Conditions associated with ageing, including chronic infectious and NCDs, as well as frailty and disability | Provides strategic direction over 5 years to promote healthy ageing and delay functional  inability among older persons, with a focus on aligning the health sector with the needs of older persons and promoting ageing-friendly environments. | The only edition of the strategy provides a strong foundation for how a multimorbidity strategy could look given that the challenges and needs of older persons are complex and often involve multiple conditions, both chronic infectious and non-communicable. |
| **Zimbabwe National Medicines Policy** | 2012 | All NCD medicines supplied in Zimbabwe | Policy to improve, within the available resources, the  health of the majority of the population of Zimbabwe by treating, curing, reducing or preventing diseases and disorders of health through medicine procurement,  promotion of local production of essential medicines, management and use. Focus on ensuring safety, affordability, and appropriate use | Not specific to NCDs or multimorbidity but provides information about key supply chain characteristics and challenges which are especially pertinent in the context of NCDs. Suggests a greater role of domestic pharmaceutical companies in ensuring more affordable and consistently available essential medicines. |

1. Banda-Mtaula, G.T., Phiri, E.R.M., Taegtmeyer, M. *et al.* An exploratory study of context and factors shaping policies for integrated management of multimorbidity in Malawi. *Health Res Policy Sys* **23**, 84 (2025). https://doi.org/10.1186/s12961-025-01358-0
