# Supplementary material for: Pathways of health care for people living with multimorbidity in two Southern African countries
Source: PLoS One. 2026 Jun 12;21(6):e0351251. doi: 10.1371/journal.pone.0351251 (PMC13262806; doi:10.1371/journal.pone.0351251)
Supplement: S2 Table — (DOCX) [file pone.0351251.s003.docx]

**S2 Table of health providers for people with multimorbidity**

**Country:**

**Date(s) of completion:**

**Name(s), and role(s) of people inputting into content of this table:**

Please complete in conjunction with the care pathway figure.

Please add as much information as possible into the table below. Add extra rows as needed.

**If supporting documents are available online, please paste weblinks here:**

| **Health provider** | **Definition (what does this include)** | **Available nationally, regionally, locally, or not available** | **Challenges/ Barriers**  **(i.e. what doesn’t work so well)** | **Solutions/ Facilitators**  **(i.e. what works well)** |
| --- | --- | --- | --- | --- |
| **PRIMARY**  Community clinics/ health centres/ polyclinics |  |  |  |  |
| **SECONDARY**  Hospital (mission/ district/ rural) |  |  |  |  |
| **TERTIARY**  Hospital (provincial) |  |  |  |  |
| **QUATERNARY**  Hospital (central) |  |  |  |  |
| Community-based services (e.g., screening, outreach, support) |  |  |  |  |
| Pharmacy  Services |  |  |  |  |
| Diagnostic Services  (e.g. laboratory / radiology) |  |  |  |  |
| NGO  services |  |  |  |  |
| CSO  services |  |  |  |  |
| Private services/ providers (including informal) |  |  |  |  |
| Traditional Healers |  |  |  |  |
