# Supplementary material for: Pathways of health care for people living with multimorbidity in two Southern African countries
Source: PLoS One. 2026 Jun 12;21(6):e0351251. doi: 10.1371/journal.pone.0351251 (PMC13262806; doi:10.1371/journal.pone.0351251)
Supplement: S3 Table — (DOCX) [file pone.0351251.s004.docx]

S3 Table showing challenges and opportunities in Malawi

| **Challenges/Barriers (what doesn’t work well)** | **Facilitators (what works well)** |
| --- | --- |
| **COMMUNITY LEVEL** | |
| **Weak information systems**   - Inadequate awareness of communities: early screening and treatment - Lack of awareness among caregivers and CHWs on NCDs - Underdeveloped health prevention services burden the care system. | **Existing support groups**   - Support groups coordinated by CHWs, usually HSAs. - Clear referral pathways |
| **Weak human resources capacity**   - Inadequate competencies for the CHW - Lack of motivation and commitment among volunteer CHWs. - Lack of specialists within proximity to district hospitals - Limited supervision from the district level due to limited resources - Shortage of health workforce - work overload on existing staff. |  |
| **Costs for ongoing care**   - Travel costs - patients not living within proximity of health facilities. - Healthcare-related costs resort to traditional/ faith healers   **Fear of discrimination**   - Fear of discrimination results in low levels of health-seeking behaviour |  |
| **Lack of knowledge including self-management**   - Self-management after hospital discharge. - Myths and misconceptions – NCDs are STIs. - Lack of knowledge about chronicity. Stop taking medication - Education materials are very few and in health centres only - Struggling to change diet and lifestyle - are materials effective? | **Peer support groups**   - Awareness of NCDs through community meetings. - Testimonies during public events. - Peer support on adherence and self-management. - Testing members and referral to health facilities. |
| **Discrimination**   - Discrimination because of misconceptions. - Special diet seen as expensive. Communities do not understand | **Personal testing equipment**   - Due to a lack of testing services at the hospital, some patients have personal BP machines and Glucometers |
| **Limitations with community structures**   - Community-Based Organisations (CBO)s focus on HIV/AIDS and other livelihood programs than NCDs. - Some communities do not have Health Surveillance Assistants (HSAs). HSAs lack knowledge. - Peer groups not recognised at health centres, affecting referrals. - Patients told to buy medicines from private pharmacies | **Supportive roles of CBOs**   - Leveraged on CBOs to mainstream NCDs and engage traditional leaders. - Livelihoods projects, i.e., nutrition rations and loans |
| **PRIMARY LEVEL** | |
| **Weak human resources capacity**   - Inadequate numbers of clinical staff who can manage NCDs. - Inadequate clinical competencies and confidence among providers - Lack of flexibility to shift to integration of NCD services | **NCD coordinators**   - Health centres have one NCD Coordinator who manages the coordination of NCDs in a particular area. |
| **Lack of equipment**   - Testing kits like glucometers. Screening only those with symptoms - Unsatisfactory/inconsistent laboratory support |  |
| **Unavailability of drugs**   - Delayed treatment. Increased patient costs. Poor patient outcomes. - Limited treatment options. Little room to adjust medications |  |
| **Weak patient referral mechanisms**   - Resulting in delayed care and increases on patient costs. |  |
| **Delayed diagnosis and referral**   - Having symptoms for over two years without testing and diagnosis. - Reliance on painkillers for relief of long-standing symptoms. - No comprehensive medical tests in rural facilities save for malaria. | **Referral of emergency cases**   - Due to delays, most patients develop stroke or severe illness. - Quick referrals are made on such context. |
| **Access to the PHC facilities**   - Long distances to nearest public health centres > ten kilometres. - Unreliable mode of transport. Push bikes for rural communities. |  |
| **SECONDARY LEVEL** | |
| **Weak human resources capacity**   - Inadequate clinical staff who can manage NCDs at Secondary facility. - Inadequate clinical competencies among providers of NCD services | **NCD coordinators and specialists**   - Coordinator responsible for NCDs in the district. - A specialist goes around the district on set days. |
| **Unavailability of drugs**   - Delayed treatment. Increased patient costs. Poor patient outcomes. - 17% of district hospitals met the requirement for HTN drugs (NCDI) | **Integration of services**   - In some hospitals, for instance Neno district hospital, NCD clinics are integrated with ART clinics. |
| **Lack of equipment**   - Testing kits like glucometers. Screening only those with symptoms - Lack of individuals who can install and run the equipment**.** |  |
| **Lack of Infrastructure**   - Patient samples sent to main hospital laboratories (Queens, KCH) - The high volume of samples results in delays and misplacement. |  |
| **Weak patient referral mechanisms**   - Resulting in delayed care and increases on patient costs. |  |
| **Underutilization of digital health platforms**   - Leading to inefficient patient management practices |  |
| **TERTIARY LEVEL** | |
| **HCW/ patient relationships**   - Some doctors unhappy with patients self-testing – patients frustrated. - Focus is on existing conditions - no efforts to test other conditions. - Long queues. HCWs overwhelmed. No comprehensive consultations - Nurses’ poor attitude. Caregivers unable to comprehend information |  |
| **Complex and protracted patient pathways**   - MM patients visit different departments. Delays and longer times - No direction to the different specialists. They explore this on their own. - Patients with MM prefer an integrated one-stop-shop clinic |  |
| **Unavailability of drugs**   - Pharmacist advising patients to buy medicines. Patients unable to - Resorting to cheaper alternative medicines or relying on painkillers. - Given expired medicines or medicines that are about to expire. |  |
| **Weak human resources capacity**   - Inadequate number of specialists to provide care as well undertake district visits. - Inadequate clinical competencies among providers of NCD services | **NCD coordinators and specialists**   - Every central hospital has 3 coordinators: Mental Health, Chronic sickness, or injuries. |
|  | **Better services at central hospitals**   - Central and district hospitals likely to have medications and equipment for NCDIs than health centres. |
