# Supplementary material for: Pathways of health care for people living with multimorbidity in two Southern African countries
Source: PLoS One. 2026 Jun 12;21(6):e0351251. doi: 10.1371/journal.pone.0351251 (PMC13262806; doi:10.1371/journal.pone.0351251)
Supplement: S4 Table — (DOCX) [file pone.0351251.s005.docx]

**S4 Table showing challenges and opportunities in Zimbabwe**

| **Health provider** | **Definition (what does this include)** | **Available nationally, regionally, locally, or not available** | **Challenges/ Barriers**  **(i.e. what doesn’t work so well)** | **Solutions/ Facilitators**  **(i.e. what works well)** |
| --- | --- | --- | --- | --- |
| **PRIMARY** | Includes health centres, clinics, rural hospitals (rural), and polyclinics (urban). Also includes private clinics and surgeries. Defined as “first and lowest level” in the Zim national health strategy | Nationally | - Medicines especially for NCDs (6% of health budget for medicines outside donor-funded programmes, even though that doesn’t mean it’s actually disbursed -Natpharm usually has no stock) - High staff attrition- understaffed - Few BP machines or glucose strips, especially in OPDs - Fragmented programme structure leading to multiple queues for MM patients and uneven resourcing/infrastructure (e.g., OI clinic much more funded than OPD) - Erratic /incomplete/non-existent M&E beyond programme-supported records hinders care continuity, leads to loss to follow up, no data for planning - User fees even for chronic patients receiving check-ups/refills (which are rarely available) | - Historically strong network of clinics, including in rural areas such that travel isn’t prohibitive for many (not without exception, e.g., the elderly, more remote areas, ‘squatter’ camps beyond formal city planning) - Some HIV-NCD integration pilots through programmes - RGNs very well trained historically - General perception that nurses are caring and compassionate (surprising given the low morale/high stress) perhaps contrasts with other settings e.g., SA, with well-documented problems with ‘crisis of care’) - EDLIZ widely used as a clinical guideline, especially at primary level, with strong adherence where resources permit - Ad hoc facility-level initiatives to ‘know’ MM among their patients (e.g., notes in registers) |
| **SECONDARY** | District hospitals and equivalents (municipal referral hospitals, mission hospitals, etc.) provide emergency, ambulatory, and inpatient care. Also includes private hospitals | Nationally, but mostly rural, besides e.g., ID hospitals in urban areas | - Medicines, especially NCDs - Staff attrition - User fees - Shortages of doctors, some have been running for years during COVID-19, without a single doctor end up referring for NCD diagnosis - Shortage of beds, sundries, commodities, lab infrastructure and reagents - Fragmented programme structure leading to multiple queues for MM patients and uneven resourcing/infrastructure (e.g., OI clinic much more funded than OPD) - DMOs (who head the hospitals) are generally thrown in at the deep end straight after basic training, having to become leaders on the job quickly. Need for greater mentoring/support and further training (e.g., MSc public health or family medicine) - Patients often referred either upward to tertiary/quat or to private sector for investigations | - Diagnosis and initiation of HPT, DM, cervical cancer, and other NCDs that can’t currently be done at primary level - Some perceptions that district-level care is pretty good for providing generalist care and isn’t usually prohibitively far from communities (with exceptions). - District hospitals the focal point for starting to train nurses at the primary level in NCD screening and treatment (there are now protocols for use of WHO PEN programme guidelines to decentralise NCD care, though still in the early days). |
| **TERTIARY** | Network of provincial hospitals (one per province except Harare/Bulawayo) provides emergency, ambulatory, and specialist inpatient services | Rural only | - Medicines, especially NCDs (see above) - Fragmented programme structure leading to multiple queues for MM patients and uneven resourcing/infrastructure (e.g., OI clinic much more funded than OPD) - Staff attrition - User fees - Shortage of beds, sundries, commodities, lab infrastructure and reagents - Often, any spare resources are used just to cater for emergencies and transport - Staff attrition and lack of post-basic training, especially among nurses, (leading to more ‘on-the-job’ training, often by - Supposed to have specialist doctors but posts not filled in many provinces, e.g., Gwanda has only a general surgeon, orthopaedic surgeon, and obstetrics & gynaecology among the furthest from Harare, no coincidence - Shortage of beds, sundries, commodities, lab infrastructure and reagents | - Some hospitals with a considerable and growing number of specialists, which favours multidisciplinary teams for more complex MM care (e.g., Marondera province nearest Harare, no coincidence) |
| **QUATERNARY** | Specialist inpatient and outpatient services as well as University teaching facilities | Urban only | - Medicines - User fees - Staff attrition - A big challenge for central hospitals is patients ‘bypassing the primary level and going straight to the central hospital, creating bottlenecks of patients - Lack of specialist treatments (e.g., limited dialysis capacity, cancer treatment, and not all surgeries that need to be performed can be) - Lack of investigations (patients sent to private labs, which are hugely expensive) - Multiple queues and often a lack of join-up between (sub-)specialist clinics (e.g., disjointed treatment plans, multiple medicine prescriptions that can’t be afforded) - Beds full of chronic / MM patients, many elderly, and geriatric care is currently underdeveloped in Zimbabwe (one of our group is the only geriatrician in Zimbabwe) | - Availability of some (sub-) specialities and efforts to create more specialist clinics where possible (e.g., endo, lipid, rheumatology) - Strong ‘generalism’ even within the specialities (e.g. any sub-specialist will also run a general clinic/ward, often alternating) - Lots of very clever people, very well trained (just not enough resources to get the most out of them) - Some MM-related research is currently being conducted, though most funding, aspirations, and initiatives generally specialist/disease-focused - Generalist training through MPH and MSc Family Med - Specialist training provided (though not sub-specialist overseas only) |
| Community-based services (e.g., screening, outreach, support) | Not formally defined as a level of care but composed mostly of network of community health workers (formerly ‘village health workers’ in rural areas in the old days) | National | - Erratic screening at best, highly dependent on donor support - Screening/outreach often ‘piggybacked’ on programme activity, e.g. transport is heading out for a programme so an opportunity for some community screening - Community health workers are overburdened with increasing numbers of programmes adding their protocols (e.g., recently CHWs in Chitungwiza were asking for double pay per screening since there are so many commitments) | - Strong network of CHWs in rural districts based on HIV platform (which was beneficial during COVID for decentralising services and domiciliary care) - Some outreach for screening of NCDs, often either ‘piggybacked’ on programme outreach activities or programme-funded where programme funding available some teams have been trained in the ‘whole package’ (HPT, DM, mental health, cervical cancer, PREP) but often overburdens CHWs. Funding is scant, currently erratic |
| Pharmacy  Services | Huge proliferation of retail pharmacies in recent years |  | - Major problem is that there is no NCD medicine funding, so the vast majority of NCD meds are prescribed in public sector for purchase at retail pharmacies - Medicines are usually charged in USD, which not all have access to and are prohibitively expensive | - Provide a vital role in providing medicines (to those who can afford) where the government cannot - Fairly strong regulatory infrastructure through MCAZ, which supports rational drug use |
| Diagnostic Services  (e.g. laboratory / radiology) | Private laboratories (e.g., Lancet) |  | Many lab investigations not available in the public sector and sent to private labs at an extortionate cost (e.g., biopsies) | Provide a vital role in providing diagnostics (to those who can afford) where the government cannot |
| NGO  services | Mostly composed of NGOs that carry out the programming of international donors (e.g., OPHID, Zim-TTECH, AHF, Newlands) | National | - Almost all are oriented toward the ‘big three’ and MNCH services - HIV exceptionalism draws away funding, government staff, and even visibility of diseases away from the core needs of the public sector, driving uneven fragmentation of health services across the country - Currently no ‘home’ for NCDs with the NGOs and donors that fund them, so they are generally slipping through the cracks - Current integrated care models (see next col) not scalable to a national (or even provincial/district) health level, isolated to a chosen defined cohort that is not even achievable within the broader HIV platform let alone nationally) | - Some NGO clinics (notably Newlands in Harare and AHF in Bulawayo) have managed to create almost model integrated MM care services for people living with HIV (‘siloes within siloes’) - Lots of interest in NCD integration, but mostly oriented towards screening, no movement currently towards funding medicines - Surveillance of NCDs/MM tends to be better within programme cohorts - Very strong M&E infrastructure which provides a model for strengthening of M&E for NCDs and less supported areas (often M&E for all areas in fact improves in facilities with programme backing) - Some NGOs doing community outreach for NCDs (e.g. Diabetic association, Sizolwethu Health Trust) |
| CSO  services | A range of CSOs throughout the country variously relevant to MM, from urban planning/governance to disease-specific organisations | Nationally (highly heterogeneous) | - Most of these are tailored towards HIV and certain vulnerable groups. - Very few groups championing NCDs/mental health, none specific to multimorbidity. - Biased towards urban settings (where they seem to have more influence) | - Many CSOs a strong presence, especially at local levels, e.g. Bulawayo United Residents’ Association have a strong voice in municipal service provision - Some CSOs within the HIV sphere are beginning to expand their remits to other health issues that intersect with HIV (Universal Health Coverage, mental health, NCDs, etc) |
| Private services/ providers (including informal) | Private surgeries, GPs, and private hospitals  Informal includes market vendors, illicit medicine wholesalers, unlicenced medical practitioners operating out of their houses or hair salons, etc. |  | - Doctors not adequately incentivised to work in public sector and those that do almost invariably have private practices too - Private doctors viewed by some in the public/NGO sectors as not well versed in public health approaches and pick and choose what to focus on (e.g., might avoid sex education or things that they feel might make the patient uncomfortable/less likely to return) - Less inclination for standardised/guideline-based approaches, and more likely to prescribe expensive medicines (which might not be indicated by EDLIZ) and investigations - Perception that many services supposed to be provided in public sector are referred to their own private practices where money can be made - Informal medicine traders often responsible for fake and substandard medicines (and these are often bought by private pharmacies and GPs so find their way into formal sector) - Often there is theft from formal facilities for sale on open market | - Often able to provide individualised (person-centred), generalist and specialist and even integrated MM care. Some doctors become known for providing care for the elderly, many have post-basic MSc family medicine or MPH qualifications (so can call themselves ‘specialist community/family physicians’) - Potentials for learning from the private sector for modelling MM care, e.g., viewed as closer to the UK GP model |
| Traditional Healers | Faith healers and traditional healers | Nationally (highly heterogenous) | Some perceptions that this leads to suspicion/poor adherence to medicines | - Accessible and affordable at the community level - Provide holistic care that is trusted by those who use their services - Existing Directorate of Traditional Healing at the MoHCC lending opportunities for formalisation and integration in NCD/MM care |
